# Characterization and Utilization of Disulfide-Bonded SARS-CoV-2 Receptor Binding Domain of Spike Protein Synthesized by Wheat Germ Cell-Free Production System

**DOI:** 10.3390/v14071461

**Published:** 2022-07-01

**Authors:** Yutaro Yamaoka, Sundararaj Stanleyraj Jeremiah, Rikako Funabashi, Kei Miyakawa, Takeshi Morita, Yusaku Mihana, Hideaki Kato, Akihide Ryo

**Affiliations:** 1 Department of Microbiology, Yokohama City University Graduate School of Medicine, Yokohama 236-0004, Japan; yamaoka-yutaro@kanto.co.jp (Y.Y.); rediffjerry@gmail.com (S.S.J.); t206518e@yokohama-cu.ac.jp (R.F.); keim@yokohama-cu.ac.jp (K.M.); t186075b@yokohama-cu.ac.jp (T.M.); mihana-yusaku@kanto.co.jp (Y.M.); 2 Life Science Laboratory, Technology and Development Division, Kanto Chemical Co., Inc., Isehara 259-1146, Japan; 3 Infection Prevention and Control Department, Yokohama City University Hospital, Yokohama 236-0004, Japan; ekato@yokohama-cu.ac.jp

**Keywords:** COVID-19, SARS-CoV-2, RBD, disulfide bond, cell-free protein synthesis, drug screening

## Abstract

The spike protein (SP) of SARS-CoV-2 is an important target for COVID-19 therapeutics and vaccines as it binds to the ACE2 receptor and enables viral infection. Rapid production and functional characterization of properly folded SP is of the utmost importance for studying the immunogenicity and receptor-binding activity of this protein considering the emergence of highly infectious viral variants. In this study, we attempted to express the receptor-binding region (RBD) of SARS-CoV-2 SP containing disulfide bonds using the wheat germ cell-free protein synthesis system. By adding protein disulfide isomerase (PDI) and endoplasmic reticulum oxidase (ERO1α) to the translational reaction mixture, we succeeded in synthesizing a functionally intact RBD protein that can interact with ACE2. Using this RBD protein, we have developed a high-throughput AlphaScreen assay to evaluate the RBD–ACE2 interaction, which can be applied for drug screening and mutation analysis. Thus, our method sheds new light on the structural and functional properties of SARS-CoV-2 SP and has the potential to contribute to the development of new COVID-19 therapeutics.

## 1. Introduction

The pandemic of the novel coronavirus disease (COVID-19) caused by SARS-CoV-2 is a serious global public health concern. Due to availability of state-of-the-art bioinformatics and molecular biological techniques, the basic biology of SARS-CoV-2 was deduced in a considerably short span of time, which helped in the development of countermeasures against this viral infection [1]. Of all the viral proteins, the most studied is the spike protein (SP) as it is the principal protein involved in viral infection. The receptor-binding domain (RBD) of the SP is the essential mediator of viral entry through the angiotensin-converting enzyme receptor 2 (ACE2) of the target cell and is exploited for the development of diagnostic and antiviral measures, including vaccines [2,3]. 

One of the key factors involved in the RBD–ACE2 interaction is the cysteine–cysteine disulfide bonds (S-S), which are responsible for the proper configuration of the RBD quaternary structure that facilitates its interaction with ACE2 [4]. Disruption of S-S on the RBD impairs its interaction with ACE2, thereby impairing viral entry [5,6,7]. In order to mimic biological processes, the S-S of RBD must be kept intact while the protein is produced in in vitro systems for diagnostic, therapeutic and research applications. 

Various protein production systems are currently available for the in vitro synthesis of proteins. The cell-based systems for this purpose comprise prokaryotes (*Escherichia coli*), yeasts, algae, insects and mammalian cell systems [8,9]. Although prokaryotic systems have the advantage of low cost and easy scalability, they have the disadvantage of improper post-translational modifications (PTM) and protein folding due to the lack of eukaryotic ribosomal machinery [10]. Insect-based systems involve a cumbersome procedure of baculovirus vector production [11]. Mammalian cell-based systems can produce excellently folded proteins with precise PTMs but have the disadvantage of demanding culture conditions and requiring long culture times as compared with prokaryotic and cell-free systems [12].

Cell-free protein expression systems offer the advantage of a simple and rapid procedure to produce good-quality proteins, retaining their biologically relevant quaternary structure [13]. One of the most well-known systems of this type is the wheat germ cell-free protein expression system (WG) [14]. However, the problem with conventional WG is the use of dithiothreitol (DTT) in the wheat germ extract and substrate solution to enhance translation efficiency [15]. DTT is known to disrupt S-S in the synthesized protein [15,16,17]. Modification of the WG (EP-WG) with the inclusion of the endoplasmic reticulum oxidoreductase-1 α (ERO1α) and protein disulfide isomerase (PDI) overcomes this drawback to produce biologically similar proteins with intact S-S [18,19,20].

In this study, we synthesized RBD with intact S-S using the EP-WG and assessed its antigenicity and binding efficiency with ACE2. We further utilized this RBD to develop an RBD–ACE2 interaction assay to screen for inhibitors of this interaction. 

## 2. Materials and Methods

### 2.1. Cells Lines and Virus

VeroE6/TMPRSS2 cells (JCRB #1819) [21] were maintained in Dulbecco’s Modified Eagle’s Medium (DMEM) containing 10% (V/V) fetal bovine serum (FBS) in 5% CO_2_ at 37 °C. SARS-CoV-2 prototype WK521 (JPN/TY-WK-521/2020, EPI_ISL_408667), Delta TY11-927 (JPN/TY11-927-P1/2021, EPI_ISL_2158617), Omicron BA.1 TY38-873 (GISAID Accession #EPI_ISL_7418017) and BA.2 TY40-385 (GISAID Accession #EPI_ISL_9595859) were obtained from the National Institute of Infectious Diseases in Japan and handled in biosafety level 3 laboratories. SARS-CoV-2 strains were propagated in VeroE6/TMPRSS2 cells cultured in DMEM supplemented with 2% FBS.

### 2.2. Plasmids Construction

Complementary DNA encoding the spike protein of SARS-CoV-2 (GenBank No. NC_045512) was chemically synthesized. RBD regions of spike proteins (319–529 aa) were amplified and inserted into pEU-MCS-s1-bls and pEU-MCS-TEV-His vectors using the In-Fusion HD Cloning kit (Takara Bio, Otsu, Japan). cDNA encoding the peptidase domain of ACE2 (GenBank No. BC039902, 19–615 aa) genes were amplified from the Mammalian Gene Collection complementary DNA library and subcloned into the pEU-MCS-flag vector. Alanine substitution mutants of RBD were generated using the PrimeSTAR Mutagenesis Basal Kit (Takara Bio, Otsu, Japan).

### 2.3. Recombinant Protein Preparation

Cell-free protein synthesis of RBD containing the disulfide bond was performed as described below, using reagents obtained from Cellfree Sciences (Yokohama, Japan). In vitro transcription was carried out using 5× Transcription Buffer LM and SP6 RNA Polymerase, according to the manufacturer’s instructions. Then, 5.5 mL of 1× SUB-AMIX SGC DTT-free was placed in a well of a 6-multi-well, flat-bottom plate. The translation mixture consisted of 250 µL of mRNA, 250 µL of WEPRO7240H, 2 µL of 1 mg/mL creatine kinase and 100 µL of PDI and ERO1α mix (recombinant protein derived from *Homo sapiens*) and was transferred into the bottom of the well containing 1× SUB-AMIX SGC DTT-free to form a bilayer. RBD was also synthesized by conventional WG, as previously described, with slight modifications [22]. To improve protein solubility, Brij-35 were added into the translation mixture at a final concentration of 0.04%. After incubation at 16 °C for 20 h, synthesized proteins were confirmed by SDS-PAGE, followed by CBB staining with Rapid CBB KANTO 3S (Kanto chemical, Tokyo, Japan) and immunoblotting. Band intensities of immunoblotting were quantified using ImageJ software version 1.49. The protein purification was carried out using Ni-Sepharose Fast Flow beads (Cytiva, Waukesha, WI, USA). Concentration of purified RBD was quantified by the TaKaRa BCA Protein Assay Kit (Takara Bio, Otsu, Japan), using bovine serum albumin as a standard. For the AlphaScreen assay, biotinylated RBD protein and flag-tagged ACE2 were produced in the presence of biotin ligase and 0.5 µM of D-biotin in the translation mixture. Recombinant RBD protein (Cat# 8COV1; Hytest, Turku, Finland) produced by the mammalian cell expression system was used as a positive control. For the binding assay of Soyasaponine I, 100 µg of recombinant RBD protein was biotinylated using Biotin Labeling Kit-NH2 (Dojindo Laboratories, Kumamoto, Japan). 

### 2.4. Clinical Specimens

Participants were recruited among healthcare workers at Yokohama City University Hospital in March 2021 [23]. Serum samples collected from the time prior to vaccination and 4 weeks after the first dose of Pfizer/BioNtech mRNA vaccine were retrieved for this study. We randomly selected a set of 40 samples and used them for ELISA analysis. This study was approved by the Institutional Review Board of Yokohama City University (Reference No. B210300001), and the protocols used in the study were approved by the ethics committee.

### 2.5. ELISA

Each recombinant RBD protein was incubated with or without 100 mM of DTT for 30 min at 37 °C and then subjected to ELISA. RBD was diluted in PBS to a concentration of 0.5 µg/mL, and then immobilized to an ELISA plate (Thermo Fisher Scientific, Rockford, IL, USA). After blocking with 3% skim milk, 100 µL of serum sample diluted with 1% skim milk (1:100) was added and incubated for 1 h. After three washes with PBS-T, 100 µL of HRP-conjugated mouse anti-human IgG antibody at a concentration of 20 ng/mL, diluted with 1% skim milk, was added into each well and incubated for 60 min at RT. After washing with PBS-T, 100 µL of TMB substrate (Kirkegaard & Perry Laboratories, Washington, DC, USA) was added and incubated for 15 min. The chromogenic reaction was stopped by adding 50 µL of 2 M H_2_SO_4_, and absorbance at 450 nm was measured on a GloMax Explorer plate reader (Promega, Madison, WI, USA)

### 2.6. K_D_ Determinations Using Bio-Layer Interferometry (BLI)

Kinetic properties were measured by bio-layer interferometry (BLI) using the Octet RED96 instrument (Sartorius AG, Gettingen, Germany), as previously described, with some modifications [24]. Briefly, a streptavidin biosensor was loaded with 20 µg/mL of biotinylated human ACE2 protein (Cat# AC2-H82E6, ACROBiosystems) for 5 min. The association of each recombinant RBD at concentrations of 1600, 800, 400, 200, 100, 50 and 25 nM was measured for 5 min, followed by a 5-min-long dissociation phase. PBS containing 0.1% BSA and 0.02% Tween 20 was used for the assay buffer. For the binding analysis of Soyasaponin I, a super streptavidin biosensor was loaded with 20 µg/mL of biotinylated ACE2 or RBD for 5 min. The association of Soyasaponin I at concentrations of 250, 125, 62.5 and 31.3 µM was measured for 3 min, followed by a 5-min-long dissociation phase. PBS containing 2% DMSO, 0.1% BSA, 0.02% Tween 20 was used for the assay buffer. All measurements were corrected for baseline drift by subtracting a reference well. Data were analyzed using a 1:1 binding model with global fitting algorithms in the ForteBio data analysis software.

### 2.7. AlphaScreen Assay

For the drug screening assay, natural compounds at a final concentration of 100 µM were mixed with crude biotinylated RBD and pre-incubated at 37 °C for 60 min. The natural compound library was obtained from TOKIWA Phytochemical (Chiba, Japan). For detection of RBD–ACE2 interaction, 0.2–0.5 µL of crude biotinylated RBD was mixed with an equal volume of flag-tagged ACE2 in 25 µL of AlphaScreen buffer (100 mM Tris–HCL (pH 8.0), 0.1% BSA, 0.01% Tween 20). After incubation at 26 °C for 60 min, 10 μL of detection mixture containing 0.04 μL streptavidin-coated donor beads and 0.04 μL anti-FLAG acceptor beads in AlphaScreen buffer was added to each well of the 384-well AlphaPlate and further incubated at 26 °C for 60 min in the dark. AlphaScreen signals were measured using the Spark plate reader (Tecan, Maennedorf, Switzerland).

### 2.8. Infection Assay

VeroE6/TMPRSS2 pre-treated with 100 µM of natural compounds or 25 µM of Camostat Mesilate (Cat# 039-17761; FUJIFILM Wako Pure Chemical, Osaka, Japan) for 3 h at 37 °C were infected with SARS-CoV-2 (MOI = 0.002–0.05) for 2 h in the presence of natural compounds. After removing the virus, the cells were further incubated with medium containing drugs. Viral RNA was isolated from 140 µL of the culture supernatant using the QIAamp Viral RNA Mini Kit (QIAGEN, Düsseldorf, Germany) after 48 h post-infection. Viral genome was quantified by one-step RT-PCR using the NIID-N2 primer set and CFX96 Touch Real-Time PCR Detection System (Bio-rad, Hercules, CA, USA). Cell viability was assessed using CellTiter-Glo (Promega, Madison, WI, USA) after 72 h post-infection.

### 2.9. Docking Simulation

The crystal structure of SARS-CoV-2 RBD bound with ACE2 (PDB code.6m0j) was used for molecular docking. The molecular docking simulations were carried out using AutoDock Vina [25]. Protein surface was visualized using the UCSF Chimera software version 1.16 (University of California, San Francisco, CA, USA) [26].

## 3. Results 

### 3.1. Wheat Germ Cell-Free System with ERO1α and PDI Produces RBD with Intact Disulfide Bonds

**Figure 1:**
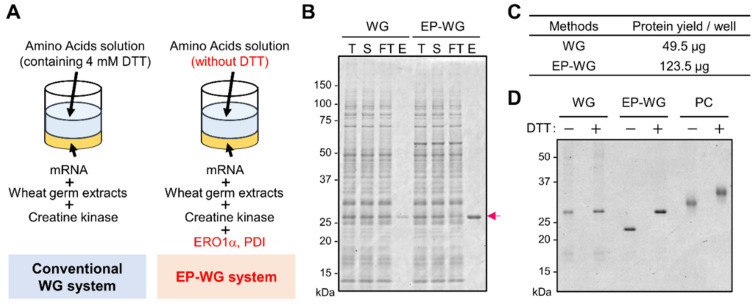
Production of SARS-CoV-2 RBD containing disulfide bonds using the wheat germ cell-free protein synthesis system. (**A**) Schematic diagram of the different wheat germ (WG) cell-free protein synthesis methods. DTT-free amino acid substrate solution and WG containing ERO1α and PDI (EP-WG) was used for the synthesis of disulfide-bond-intact protein. (**B**) Purification of RBD using nickel-chelated sepharose beads. Each protein fraction was analyzed by SDS-PAGE and visualized by CBB staining. Arrow indicates the RBD. T, Total; S, Supernatant; FT, Flow Through; E, Elution. (**C**) Comparison of protein yield synthesized by two methods. Protein yield was normalized by expression scale (1 well of 6-multi-well plate). Protein concentrations of purified protein were quantified by bicinchoninic acid (BCA) assay. (**D**) Confirmation of disulfide bond formation of RBD using non-reducing SDS-PAGE analysis. Each protein was incubated with or without 100 mM of DTT for 30 min at 37 °C prior to SDS-PAGE analysis. Commercially available RBD protein produced by mammalian cell expression system was used as positive control (PC).

Reagent modification in the EP-WG system enables the production of proteins with intact S-S [18]. We created SARS-CoV-2 RBD by both the conventional WG and the reagent-modified EP-WG (Figure 1A). The protein yield upon purification was better in EP-WG compared to the conventional WG (Figure 1B). Protein quantification by bicinchoninic acid (BCA) assay showed a more than two-fold-higher yield of purified protein with the EP-WG over the conventional WG (Figure 1C). Non-reducing SDS-PAGE with or without DTT confirmed the presence of S-S in EP-WG-derived RBD, denoted by the difference in band size in the absence of DTT, while the band was similar to that of WG in the presence of DTT (Figure 1D). The positive control, mammalian cell-expressed RBD, showed a similar difference in band size in DTT-treated versus untreated samples, showing that the difference in band size was indeed due to the destruction of S-S by DTT. These results suggest that EP-WG could synthesize a good yield of RBD with intact S-S. 

### 3.2. RBD with Intact Disulfide Bonds Bind with Vaccine-Derived Antibodies and ACE2

**Figure 2:**
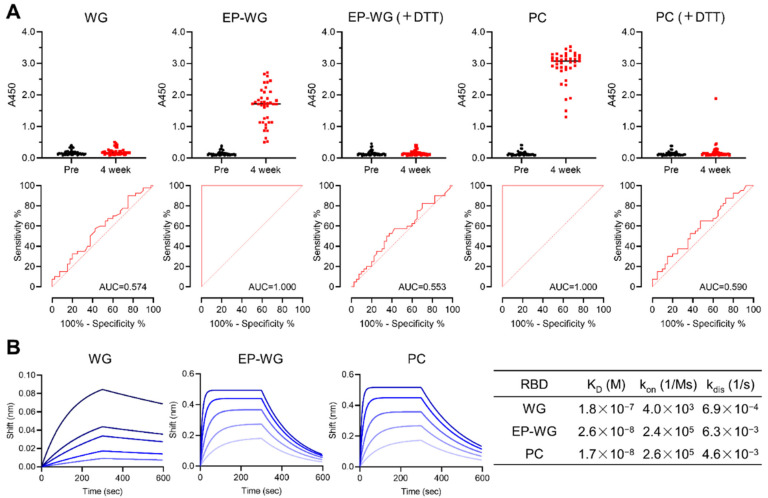
Characterization of disulfide-bonded RBD protein synthesized by wheat germ cell-free production system. (**A**) Antigenicity of disulfide-bonded RBD protein produced by wheat germ cell-free protein synthesis system with ERO1α and PDI (EP-WG). Each protein was incubated with or without 100 mM of DTT for 30 min at 37 °C and then subjected to ELISA using pre- or post-vaccinated serum (4 weeks after vaccination). The commercially available RBD protein produced by the mammalian cell expression system was used as a positive control (PC). The receiver operator characteristic (ROC) curves were constructed and the areas under the curves (AUCs) were calculated. (**B**) The K_D_ of each RBD against ACE2. Biotinylated ACE2 was immobilized on the streptavidin biosensor and then analyzed for association with each RBD protein at various concentrations (1600, 800, 400, 200 and 100 nM for WG, and 400, 200, 100, 50 and 25 nM for EP-WG and PC) by OctetRED96 instrument.

We then devised an indirect ELISA system to study the antigenicity/immune reactivity of the synthesized samples. RBD produced by different methods were allowed to react with sera obtained from pre-vaccinated immune-naive individuals and four weeks after vaccination (*n* = 40). S-S containing RBD derived from EP-WG showed robust binding with the antibodies in vaccine sera comparable to mammalian cell-derived RBD (Figure 2A). Area under the curve (AUC) calculations revealed the highly specific binding of EP-WG-derived RBD with vaccine antibodies, similar to the positive control. Conventional WG-derived RBD showed poor reactivity to antibodies, and a similar phenomenon was observed when the S-S of EP-WG- or mammalian cell-derived RBD were treated with DTT (Figure 2A). ACE2-binding affinity was calculated for the RBD synthesized by different methods. We observed high dissociation constant (K_D_) values for RBD devoid of S-S obtained from the conventional WG and low K_D_ values for RBD with intact S-S derived from the EP-WG and mammalian cell expression system (Figure 2B). These findings suggest that intact S-S is essential for RBD to bind with vaccine-derived antibodies and ACE2 receptor. 

### 3.3. AlphaScreen-Based RBD–ACE2 Interaction Assay Using EP-WG-Derived RBD

**Figure 3:**
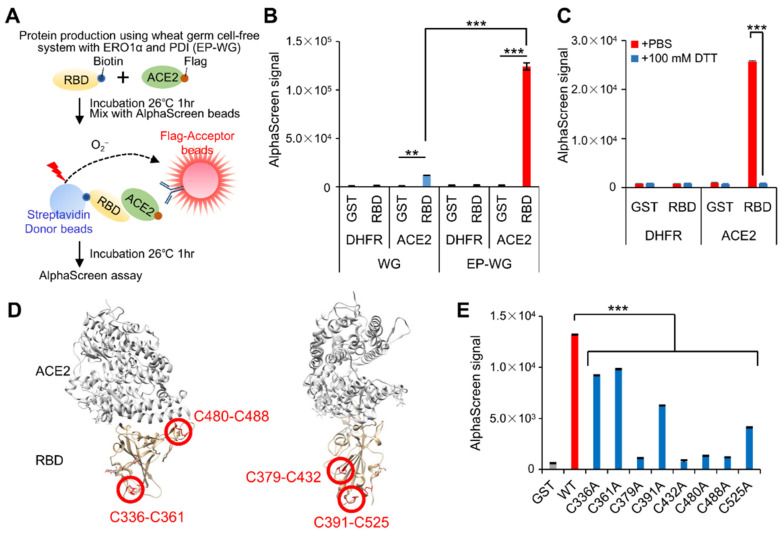
Development of AlphaScreen assay to evaluate the RBD–ACE2 interaction. (**A**) Schematic representation of AlphaScreen assay to measure the binding between biotinylated RBD and flag-tagged ACE2 produced by wheat germ cell-free protein synthesis system with ERO1α and PDI (EP-WG). (**B**) Comparison of AlphaScreen signal of each protein synthesized by two different methods. Biotinylated GST and flag-tagged DHFR were used as negative controls. AlphaScreen signal was normalized by relative protein amounts. (**C**) Disulfide-bond-containing RBD protein was incubated with or without 100 mM of DTT for 30 min at 37 °C prior to AlphaScreen assay. (**D**) Localization of disulfide bonds in the RBD protein in the tertiary structure of the RBD bound to ACE2. Disulfide bonds in the SARS-CoV-2 RBD are shown as sticks and indicated by red circles. The protein structure was obtained from PDB ID 6m0j. (**E**) Mutation analysis of cysteine residues of RBD for ACE2 interaction. AlphaScreen assay was carried out using RBD possessing alanine substitution in each cysteine residue. AlphaScreen signal was normalized by relative protein amounts. WT, Wild Type. All graph data are presented as mean ± SD, Welch‘s *t* test (two-tailed), ** *p* < 0.01, *** *p* < 0.001.

The AlphaScreen assay is a proximity-based luminescence assay that detects protein–protein interactions [27]. Interaction of RBD with ACE2 in the AlphaScreen system results in the emission of luminescence that can be quantified (Figure 3A). The assay was standardized with both WG- (devoid of S-S) and EP-WG (intact S-S—derived RBD. As expected, the EP-WG RBD produced higher luminescence, suggesting binding with ACE2 (Figure 3B and Figure S1A). The WG RBD emitted only a weak luminescence. DTT treatment of EP-WG-derived RBD abolished the luminescence production (Figure 3C).

Previous crystal structure analysis of RBD revealed four pairs of S-S occurring between eight cysteine moieties on the tertiary structure of RBD (Figure 3D) [28]. Using EP-WG, we generated eight mutant RBD each with single point mutations, replacing each of the cysteine moieties with alanaine to eliminate the S-S at the corresponding site. When subjected to the AlphaScreen assay, all the point mutants showed a significant reduction in luminescence, with some of the mutants showing a drastic reduction similar to the negative control (Figure 3E and Figure S1B). The mutations on Cys379-Cys432 and Cys480-488, which are essential for ACE2 interaction [5,29], exhibited remarkably lower signals. These results confirm the importance of S-S in the RBD–ACE2 interaction and imply that the AlphaScreen assay can be a sensitive and pliable tool to identify this interaction.

### 3.4. Drug Screeening Assay to Identify Inhibitors of RBD–ACE2 Interaction

**Figure 4:**
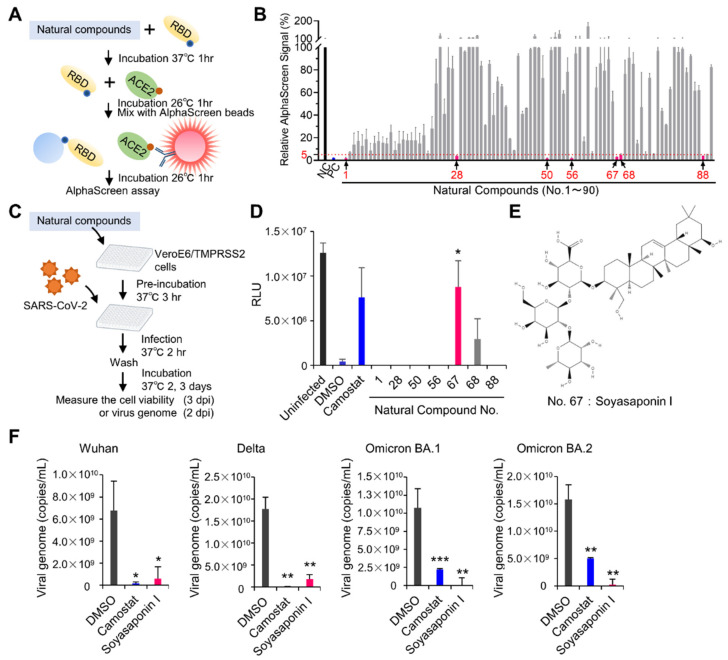
Application for drug screening using AlphaScreen assay to evaluate RBD–ACE2 interaction. (**A**) Schematic representation of drug screening using AlphaScreen assay. Ninety natural compounds were used for drug screening to inhibit RBD–ACE2 interaction in vitro. (**B**) AlphaScreen assay for drug screening. The relative AlphaScreen signal was calculated with DMSO as 100%. DTT was used as positive control (PC). Seven compounds with signals decreased to 5% or less were used for further screening. (**C**) Schematic diagram of infection assay to assess the antiviral effect of selected seven compounds. Antiviral activity of drug at a concentration of 100 µM was evaluated using cell viability as an indicator after virus infection. dpi, days post-infection. (**D**) Result of infection assay. Here, 25 µM of Camostat was used as a positive control. (**E**) Structure of drug No. 67: Soyasaponin I (PubChem Identifier: CID 122097). (**F**) Inhibitory activity of Soyasaponin I at a concentration of 100 µM against different SARS-CoV-2 strains in infection assay. All graph data are presented as mean ± SD, Welch‘s *t* test (two-tailed), * *p* < 0.05, ** *p* < 0.01, *** *p* < 0.001.

Since the AlphaScreen is a high-throughput platform, we used this assay employing EP-WG-derived RBD to identify the inhibitors of the RBD–ACE2 interaction that can serve as potential candidate drugs against SARS-CoV-2 infection (Figure 4A). We screened a library of 90 natural compounds and identified seven compounds effectively inhibiting the RBD–ACE2 interaction, with less than 5% of the signal as compared with the negative control (Figure 4B). We then tested these seven compounds in an infection assay to assess their ability to prevent infection. Each of the compounds was pre-incubated with infection-susceptible cells for three hours, following which the cells were infected with SARS-CoV-2 (Figure 4C). Of the seven compounds, only one (compound No.67) had a low cytotoxicity profile at higher concentrations (Figure S2) and effectively inhibited virus infection-mediated cell death (Figure 4D). The compound was Soyasaponin I (Figure 4E) and computational docking analysis and molecular binding analysis using bio-layer interferometry revealed that Soyasaponin I binds to RBD and possibly inhibits the interaction due to the location (Figure S3A–C). Soyasaponin I exerted its inhibitory activity on the Wuhan strain of SARS-CoV-2 and its variants Delta, Omicron BA.1 and BA.2, suggesting the effectiveness of Soyasaponin I against the epidemiologically relevant strains of SARS-CoV-2 (Figure 4F). 

## 4. Discussion

The RBD of SP is the key viral protein responsible for the infectivity of SARS-CoV-2. The major neutralizing epitopes are also present on the RBD [30]. Hence, in vitro methods to generate this protein for diagnostic, therapeutic or research purposes must ensure that the synthesized protein is identical to its natural counterpart and performs the intended biological function. The most common method for protein synthesis is the *Escherichia coli* expression system. The cytoplasm of *Escherichia coli* is under reducing conditions, and consequently RBD aggregates in the inclusion body [10]. Mammalian cell expression systems can synthesize proteins with precise PTMs and proper folding containing disulfide bonds, but require prolonged incubation times of 5–7 days or longer [12]. The WG cell-free protein expression system is considered as a simple and efficient alternative to the mammalian cell system but produces RBD devoid of S-S, which is essential for its biological activity. We have shown that the EP-WG system overcomes this drawback to produce functionally relevant RBD with intact S-S within 1 day of incubation.

Conventionally, DTT is used for the preservation of the translation efficiency of wheat germ cell extract during storage and the translation reaction [31]. However, we found that the EP-WG system devoid of DTT gave a higher yield of functionally relevant RBD than the conventional WG comprising DTT (Figure 1C). This might be due to the problem of Ni-sepharose purification for conventional WG-produced RBD, since the intensity of the total and supernatant fractions is similar to the flow-through fraction (Figure 1B). It is possible that RBD synthesized by conventional WG do not form S-S and thus aggregate or denature, and the His tag is not exposed to the protein surface. 

Although EP-WG produced functional RBD with intact S-S, it had a different band shape compared to the RBD produced by the mammalian cell expression system (Figure 1D). This is probably due to glycosylation or other PTMs that can occur in the latter system. The lack of glycosylation is one of the drawbacks of the WG system. RBD has two glycosylation sites, N331 and N343. In the current study, RBD synthesized by EP-WG had slightly lowered affinity for ACE2 than RBD produced in the mammalian cell expression system. On the other hand, Qianqian Li et al. showed that glycosylation mutants of N331 and N343 had low infectivity in pseudovirus infection experiments [32]. The C-type lectin receptor functions as an attachment receptor by binding to the glycan of the spike protein [33,34]. Therefore, RBD prepared by EP-WG might yield different results from those expressed by mammalian cells when performing cellular binding assays. In addition, some human monoclonal antibodies have been reported to recognize the epitope with glycan [35]. The presence or absence of glycans possibly affects the reactivity in the serological assay.

Cysteine residues in RBD are conserved among SARS-CoV-2 variants and SARS-related coronaviruses and are responsible for the stabilization of the RBD structure and modulating immunogenicity [5,36]. Previous studies have reported that unfolded RBD or the short form of RBD (318–510 aa; lack of Cys521) induce lower titers of neutralizing antibodies [37,38]. The majority of neutralizing antibodies against RBD induced by SARS-CoV-2 infection are antibodies to conformational epitopes [37]. mRNA vaccines induce neutralizing antibodies against RBD of conformational epitopes as well as natural infection [39]. RBD have few detectable linear epitopes in both infected and vaccinated individuals [40,41,42]. In this study, the vaccine-derived antibodies did not react with RBD whose conformation had been disrupted with DTT treatment, demonstrating that the disulfide bonds in the RBD are essential for the antigenicity of RBD. Our method to produce properly folded RBD would be a useful tool to study the immunogenicity and antigenicity of RBD for vaccine development. 

The AlphaScreen assay developed using the EP-WG-derived RBD enabled the high-throughput screening of a natural compound library to identify agents that can inhibit RBD–ACE2 interaction. We identified Soyasaponin I as a potent inhibitor of RBD–ACE2. Soyasaponin and other saponins are known to possess in vitro antiviral activity against herpes simplex virus type 1, human cytomegalovirus, influenza virus and human immunodeficiency virus type 1 [43,44]. Licorice-saponin A3 and Esculeoside A, a type of saponin, have been demonstrated to bind to SARS-CoV-2 RBD and have antiviral activity [45,46]. In the current study, the docking simulation and binding assay showed that Soyasaponin I bound to the RBD with relatively low affinity as compared with a previously reported inhibitor [47]. Licorice-saponin A3 suppresses SARS-CoV-2 infection by not only inhibiting RBD–ACE2 interaction but also inhibiting non-structural protein 7 [45]. Soyasaponin I induces macroautophagy by inhibiting the Akt signaling pathway [48]. SARS-CoV-2 promotes infection by regulating cellular metabolism and evading autophagy in infected cells [49]. Soyasaponin I possibly inhibits infection via the inhibition of nonstructural proteins or induction of autophagy. Further studies are warranted to determine the inhibitory mechanism of Soyasaponin I on SARS-CoV-2.

The rapid and worldwide outbreak of SARS-CoV-2 has led to the emergence of various mutant strains, including Omicron and its subtypes. These variants have mutations in the RBD and have acquired higher binding affinity to ACE2 and evasion ability from neutralizing antibodies [50,51,52]. It is essential to rapidly prepare functional RBD to characterize the sequential emergence of mutant strains. We have established a rapid method to synthesize biologically relevant RBD using a cell-free system and demonstrated that it is applicable to test serum antibody titers, perform mutation analysis of RBD and screen drugs targeting the RBD–ACE2 interaction. Our method would be instrumental in the antigenic and functional analysis of RBD derived from emerging variants for the development of COVID-19 therapeutics and vaccines.

## Data Availability

Original/source data of Figure 4B and Figure S3 in the paper have been deposited at Mendeley Data: https://data.mendeley.com/datasets/p4jgk48y4j/1 (Version 1, published on 30 May 2022).

## Supplementary Materials

The following supporting information can be downloaded at: https://www.mdpi.com/article/10.3390/v14071461/s1. Figure S1: Confirmation of protein expression utilized in Figure 3. Figure S2: Cytotoxicity of selected natural compounds. Figure S3: Binding analysis of Soyasaponin I against RBD. Table S1: The list of compounds screened by the AlphaScreen assay.
