# Supplementary material for: Characterization and Utilization of Disulfide-Bonded SARS-CoV-2 Receptor Binding Domain of Spike Protein Synthesized by Wheat Germ Cell-Free Production System
Source: Viruses. 2022 Jul 1;14(7):1461. doi: 10.3390/v14071461 (PMC9321213; doi:10.3390/v14071461)
Supplement: Supplementary file 1 [file viruses-14-01461-s001.zip › viruses-1778966-supplementary.pdf]

Supplementary Materials

Characterization and utilization of disulfide-bonded SARS-CoV-2 receptor binding domain of spike protein synthesized by wheat germ cell-free production system

Yutaro Yamaoka, Sundararaj Stanleyraj Jeremiah, Rikako Funabashi, Kei Miyakawa, Takeshi Morita, Yusaku Mihana, Hideaki Kato, and Akihide Ryo

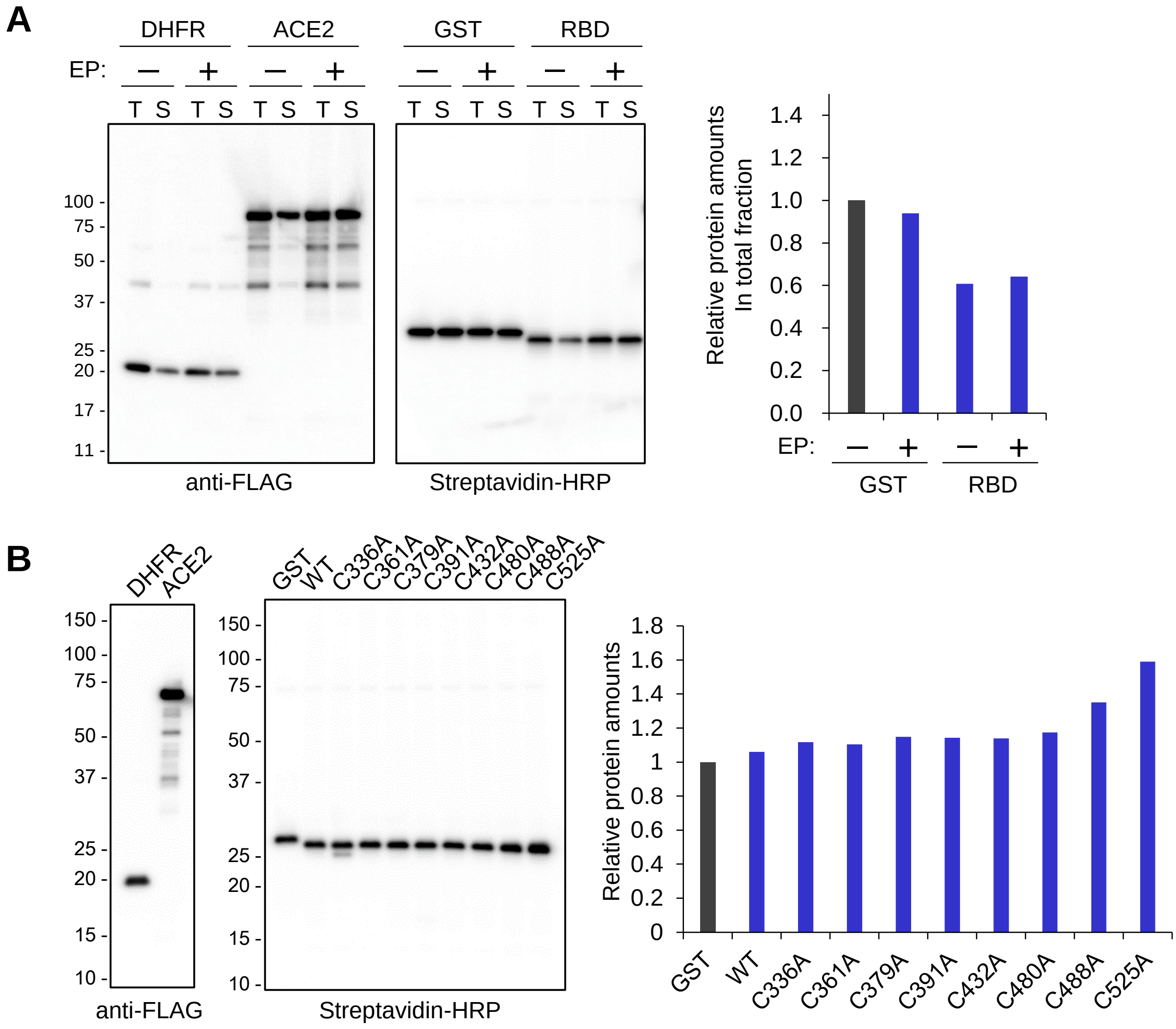

Figure S1 (Related to Figure 3): Confirmation of protein expression utilized in Figure 3.
(A, B) Each protein expression was confirmed by immunoblotting using indicated antibodies. Bar charts indicate the relative protein amounts as determined by band intensity. T, Total fraction; S, Supernatant; EP, ERO1α and PDI.

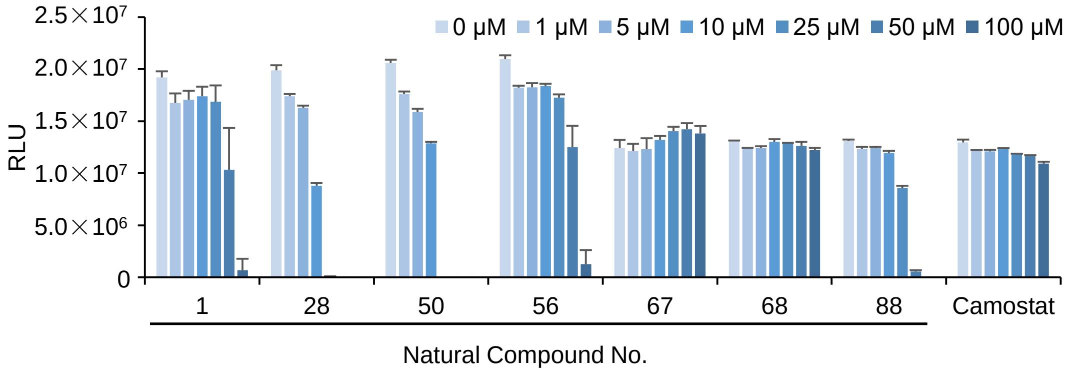

Figure S2 (Related to Figure 4): Cytotoxicity of selected natural compounds.
Cell viability of VeroE6/TMPRSS2 cells treated with seven compounds and Camostat at indicated concentrations for 48 hrs.

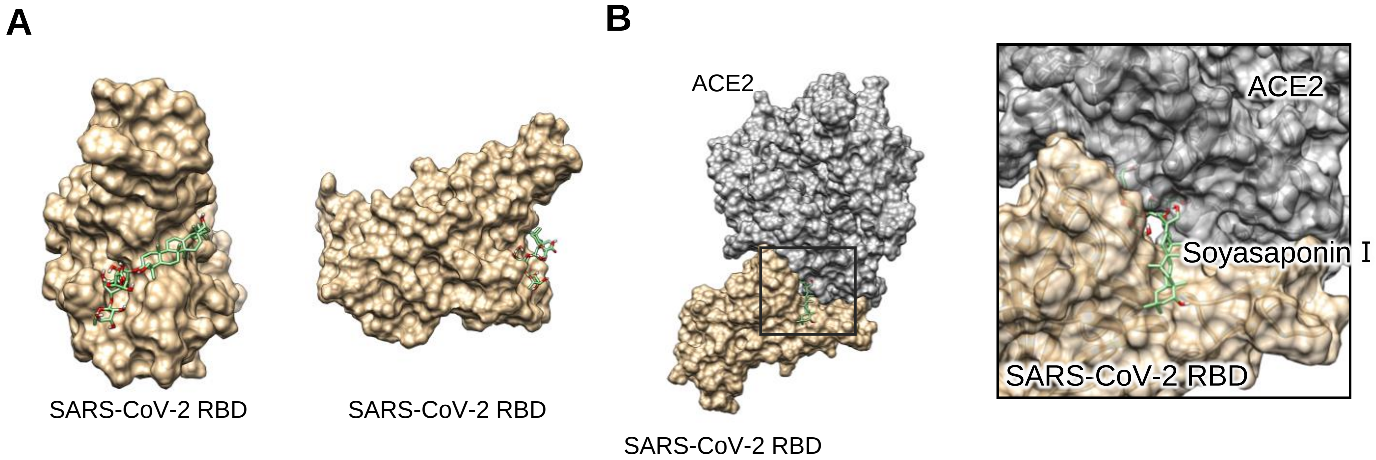

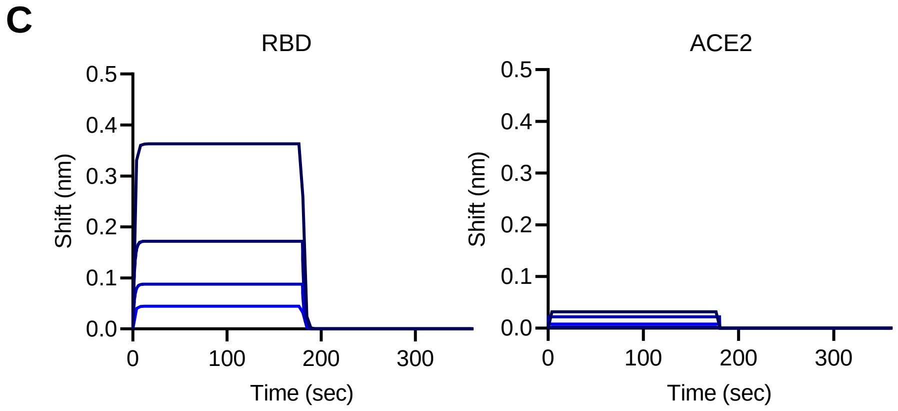

	K_D (M)	k_{on} (1/Ms)	k_{dis} (1/s)
RBD	8.2×10^{-6}	6.9×10^{4}	5.6×10^{-1}
ACE2	N.D.	N.D.	N.D.

Figure S3 (Related to Figure 4): Binding analysis of Soyasaponin I against RBD.
(A, B) Docking simulation analysis of Soyasaponin I against RBD. The protein structure was obtained from PDB ID 6m0j. Binding of Soyasaponin I to RBD surface was simulated at a docking score of -7.7 kcal/mol using AutoDock Vina. (C) Binding assay of Soyasaponin I against RBD. Biotinylated RBD or ACE2 was immobilized on the super streptavidin sensor and then analyzed for association with Soyasaponin I at various concentrations (250, 125, 61.3, 31.3 μM) by OctetRED96 instrument. The K_D of Soyasaponin I for ACE2 was not determined due to poor curve fitting (N.D.).

Table S1 The list of compounds screened by the AlphaScreen assay.

No.	Name of natural compounds
1	Delphinidin 3-glucoside chloride
2	Cyanidin 3-glucoside chloride
3	Petunidin 3-glucoside chloride
4	Peonidin 3-glucoside chloride
5	Delphinidin 3-rutinoside chloride
6	Cyanidin 3-rutinoside chloride
7	Delphinidin chloride
8	Cyanidin chloride
9	Cyanidin 3-sophoroside chloride
10	Cyanidin 3-(2G-glucosylrutinoside) chloride
11	Delphinidin 3-galactoside chloride
12	Cyanidin 3-galactoside chloride
13	Petunidin 3-galactoside chloride
14	Peonidin 3-galactoside chloride
15	Malvidin 3-galactoside chloride
16	Pelargonidin 3-glucoside chloride
17	Delphinidin 3－sambubioside chloride
18	Delphinidin 3,5-diglucoside chloride
19	Cyanidin 3-sambubioside-5-glucoside chloride
20	Cyanidin 3-sambubioside chloride
21	Delphinidin 3-arabinoside chloride
22	Procyanidin B-1
23	Hyperoside
24	Tectoridin
25	Tectorigenin
26	6-Hydroxygenistein 6,7-diglucoside
27	Tectorigenin 7-o-xylosylglucoside
28	Licochalcone A
29	Daidzin
30	Daidzein
31	Glycitin
32	Glycitein
33	Genistin
34	Genistein
35	Xanthohumol
36	Isoquercitrin
37	Luteolin
38	(+)-Catechin
39	(-)-Epicatechin
40	(-)-Epigallocatechin
41	(-)-Epicatechin gallate
42	(-)-Epigallocatechin gallate
43	3,5,7,3',4'-Pentamethoxyflavone
44	5,7,4'-Trimethoxyflavone
45	5,7-Dimethoxyflavone
46	3,5,7-Trimethoxyflavone
47	3,5,7,4'-tetramethoxyflavone
48	Liquiritin
49	Liquiritigenin
50	Glabridin
51	Hesperidin
52	Neohesperidin
53	Calycosin 7-O-Glucoside
54	Quercetin 3-O-[2''-O-(6'''-O-p-Coumaroyl)-b-D-Glucopyranosyl]-a-L-Rhamnopyranoside
55	Quercetin 3-O-[2''-O-b-D-Glucopyranosyl]-a-L-Rhamnopyranoside
56	(-)-Gallocatechin 3-O-(3''-O-methyl)gallate
57	Isoliquiritin
58	Ganoderic acid A
59	Corosolic acid
60	Soyasapogenol B
61	18β-Glycyrrhetinic acid
62	Bilobalide
63	Ginkgolide A
64	Ginkgolide B
65	Ginkgolide C
66	Glycyrrhizinic acid (Glycyrrhizin)
67	Soyasaponin Ⅰ
68	Soyasaponin Ⅴ
69	Deacylgymnemic acid
70	Ginsenoside Rg1
71	Tenuifolin
72	Withaferin A
73	Pteropodine(Uncarine C)
74	Isopteropodine(Uncarine E)
75	Rhynchophylline
76	Verbascoside (acteoside)
77	Arctiin
78	Echinacoside
79	Isoacteoside
80	6-Gingerol
81	8-Gingerol
82	10-Gingerol
83	6-Shogaol
84	Cryptochlorogenic acid
85	Neochlorogenic acid
86	Isochlorogenic acid A
87	Isochlorogenic acid B
88	Sennoside A
89	Sennoside B
90	Mangiferin
